# In infertile women, cells from *Chlamydia trachomatis *infected site release higher levels of interferon-gamma, interleukin-10 and tumor necrosis factor-alpha upon heat shock protein stimulation than fertile women

**DOI:** 10.1186/1477-7827-6-20

**Published:** 2008-05-20

**Authors:** Pragya Srivastava, Rajneesh Jha, Sylvette Bas, Sudha Salhan, Aruna Mittal

**Affiliations:** 1 Institute of Pathology – ICMR, Safdarjung Hospital Campus, Post Box no. 4909, New Delhi-110 029, India; 2 Division of Rheumatology, Department of Internal Medicine, University Hospital, 1211 Geneva 14, Switzerland; 3 Department of Gynecology & Obstetrics, Safdarjung Hospital, New Delhi-110 029, India

## Abstract

**Background:**

The magnitude of reproductive morbidity associated with sexually transmitted Chlamydia trachomatis infection is enormous. Association of antibodies to chlamydial heat shock proteins (cHSP) 60 and 10 with various disease sequelae such as infertility or ectopic pregnancy has been reported. Cell-mediated immunity is essential in resolution and in protection to Chlamydia as well as is involved in the immunopathogenesis of chlamydial diseases. To date only peripheral cell mediated immune responses have been evaluated for cHSP60. These studies suggest cHSPs as important factors involved in immunopathological condition associated with infection. Hence study of specific cytokine responses of mononuclear cells from the infectious site to cHSP60 and cHSP10 may elucidate their actual role in the cause of immunopathogenesis and the disease outcome.

**Methods:**

Female patients (n = 368) attending the gynecology out patient department of Safdarjung hospital, New Delhi were enrolled for the study and were clinically characterized into two groups; chlamydia positive fertile women (n = 63) and chlamydia positive infertile women (n = 70). Uninfected healthy women with no infertility problem were enrolled as controls (n = 39). cHSP60 and cHSP10 specific cytokine responses (Interferon (IFN)-gamma, Interleukin (IL)-10, Tumor Necrosis Factor (TNF)-alpha, IL-13 and IL-4) were assessed by ELISA in stimulated cervical mononuclear cell supernatants.

**Results:**

cHSP60 and cHSP10 stimulation results in significant increase in IFN-gamma (P = 0.006 and P = 0.04 respectively) and IL-10 levels (P = 0.04) in infertile group as compared to fertile group. A significant cHSP60 specific increase in TNF-alpha levels (P = 0.0008) was observed in infertile group as compared to fertile group. cHSP60 and cHSP10 specific IFN-gamma and IL-10 levels were significantly correlated (P < 0.0001, r = 0.54 and P = 0.004, r = 0.33 respectively) in infertile group.

**Conclusion:**

Our results suggest that exposure to chlamydial heat shock proteins (cHSP60 and cHSP10) could significantly affect mucosal immune function by increasing the release of IFN-gamma, IL-10 and TNF-alpha by cervical mononuclear cells.

## Background

Sexually transmitted *Chlamydia trachomatis *infection is an important public-health concern with major burden on female reproductive tract [1]. Untreated chlamydial infection can lead to pelvic inflammatory disease (PID) in 10% to 40% of affected women, which can result in infertility, ectopic pregnancy and chronic pelvic pain [2].

Immune responses to *C. trachomatis *60-kDa heat shock protein (cHSP60) has been associated with the pathogenesis of *C. trachomatis *associated ectopic pregnancy and tubal infertility [3,4]. A recent report from our lab suggests that detection of anti-cHSP60 antibodies would help in the early prognosis of immunopathological sequelae in *C. trachomatis *infected women [5]. The stress response in *Chlamydia *reticulate bodies is characterized by cHSP60 induction and by reduction in major outer membrane protein and lipopolysaccharide (LPS) levels, as shown in an in vitro model of persistent infection [6,7]. This stress response is believed to interrupt the normal progression of reticulate bodies to infectious elementary bodies, resulting in a longer-term persistent infection. Such persistent infections may serve as antigenic reservoirs for potentially immunopathogenic anti-cHSP immune system responses [8]. The chlamydial 10-kDa heat shock protein (cHSP10) is genetically linked to cHSP60; the two proteins bind to each other and prevent incorrect protein folding and denaturation. Thus, the pathogen's ability to survive stressful environmental conditions and persist in the host is maximized by cHSP60-cHSP10 expression.

The development of infertility is reported due to enhanced immune responses to *C. trachomatis *[9,10]. cHSP60 and cHSP10 antibodies seem to perform well in predicting tubal factor infertility (TFI) [11-17]. Cell-mediated immune responses to cHSP60 were demonstrated in women with PID and TFI [18-23]. Thus, immunopathogenesis of TFI also involves cell-mediated mechanisms. However, these studies were restricted to the peripheral immune responses. A recent study suggests that mucosal immune responses are better to predict pathogenesis as cervical cells are the actual cells encountering the pathogen [24]. In the previous report from our lab cHSP60 and cHSP10 specific proliferative responses were evaluated and suggested the probable role of cHSPs in modulation of mucosal immune responses [25]. Overall these studies suggest cHSPs specific cell mediated immune responses plays an important role in the immunopathogenesis associated with chlamydial infection. Hence it might be possible that cytokines released by cervical mononuclear cells that are in direct contact with the pathogens and with cHSPs may play a crucial role in the modulation of mucosal immune responses leading to pathogenesis. Therefore, the objective of this study was to characterize the cHSP60 and cHSP10 specific cytokine responses by cervical mononuclear cells among groups of women representing different clinical conditions, i.e. chlamydia positive fertile and infertile women for understanding their role in modulation of immune responses at the site of infection. The production of T helper type 1 (Th1)/Th2 cytokines was investigated as well as those of Tumor Necrosis Factor-alpha (TNF-α) and Interleukin (IL)-10. The role of Th1/Th2 responses in the genital tract during *Chlamydia *infection is considered to be crucial for controlling the duration of infection and subsequent tubal pathology. Indeed, Th1 cells produce Interferon-gamma (IFN-γ) that promotes the destruction of *Chlamydia *[6] but can also promote inflammatory damage and fibrosis [26] whereas Th2 cells produce IL-4, IL-5, and IL-13 believed to be critical for defense against extracellular pathogens. The production of TNF-α and IL-10 was examined because their levels have been reported to be high in cervical secretions of *C. trachomatis *infected infertile women [27].

## Methods

### Study population

A total of 368 patients attending the gynecology out patient department, Safdarjung Hospital, New Delhi, India for gynecological complaints (cervical discharge, cervicitis and infertility) were enrolled in the study. The study received approval by the hospital's ethics review committee. Thirty-nine healthy age-matched controls attending the family-planning department for birth-control measures and with no previous history of any sexually transmitted disease (STD) were also enrolled. At recruitment, a detailed clinical questionnaire was administered to each patient for collecting information on reasons for referral, gynecology history including menstruation, symptoms of genital and urinary tract infection, obstetric and medical histories. Patients with positive urine pregnancy test, recent antibiotic therapy and history of recently treated sexually transmitted infection (STI) and genital tuberculosis were excluded from the study.

### Collection of samples

The vulva was examined for lesions and vaginal/cervical discharge. The cervix was inspected for ulcers, warts, ectopy, erythema, discharge or any other abnormalities. After cleaning the endocervix with a cotton swab (Hi Media, Mumbai, India), endocervical swabs were collected from patients for diagnosis of *C. trachomatis *and other STI pathogens.

The cervical canal was wiped clean, and a cytobrush was placed within the endocervical canal so that cells from the endocervical region and the zone between the endocervical and ectocervical regions (transformation zone) could be obtained. The cytobrush was then held in a sterile centrifuge tube containing phosphate buffered saline (PBS) (pH 7.2) supplemented with 100 U penicillin/ml, 100 μg streptomycin/ml, and 100 μg glutamine/ml. All cytobrush samples had negative results for blood contamination.

### Laboratory diagnosis

Spots were made on glass slides from cervical swab samples. These were stained with fluorescein isothiocyanate conjugated monoclonal antibodies to *C. trachomatis *major outer membrane protein (MOMP) using *Chlamydia trachomatis *Direct Specimen Test kit (Microtrak, Syva Corporation, Palo Alto, CA, USA) according to the manufacturer's instructions. A sample was considered to be positive when at least 10 elementary bodies were detected. Samples with greater than one and less than 10 EBs were confirmed for positivity by polymerase chain reaction (PCR) analysis using a primer specific for 200 base pair (bp) plasmid of *C. trachomatis *[28]. Diagnosis for other STD pathogens were done by culture for *Neisseria gonorrhoeae, Mycoplasma hominis*, *Ureaplasma urealyticum *and by microscopy on gram stained smears for *Candida sps.*, bacterial vaginosis, *Trichomonas vaginalis *as mentioned earlier [27].

### Cloning of cHSP60 and cHSP10

Full-length cHSP60 gene (GenBank accession no. M58027) was amplified using unique primer set (Forward primer – 5'-TCCCcccgggATGGTCGCTAAAAACATTAAA-3' and Reverse primer 5'-ACGCgtcgacTTAATAGTCCATTCCTGCGCC-3' with restriction endonuclease sites, XmaI and SalI respectively at 5'-end). Oligonucleotides used as primers were synthesized by Microsynth, Balgach, Switzerland. PCR products were initially cloned into pGEM-T Easy vector (Promega, Madison, Wisconsin) and checked for their correct reading frame by sequencing. The cloned inserts were then excised by digestion with restriction enzymes XmaI and SalI (New England Biolabs) and ligated to the compatible sites of the expression vector pQE-60 (Qiagen Inc., Chatsworth, California) in frame with a His6 affinity tag-coding sequence at the 3' terminus. The resulting plasmids were introduced into *Escherichia coli *M15 cells and routinely grown in the presence of ampicillin (100 μg/ml) and kanamycin (25 μg/ml) (Sigma).

Cloning of cHSP10 and large-scale expression and purification of chlamydial recombinant proteins was performed as mentioned earlier [29]. Proteins under native conditions were purified by nickel chelate affinity chromatography (Qiagen Ni-NTA Superflow resin). The protein concentration was determined with the Bradford assay (Sigma). We controlled for the non-specific effects of LPS contamination by treating recombinant proteins with polymyxin B (Sigma-Aldrich). Purified proteins were subsequently characterized by SDS-PAGE and immunoblotting using monoclonal antibody against chlamydia (Alexis Biochemicals, Lausen, Switzerland), separated into aliquots and frozen at -80°C.

### Purification and culture of cervical mononuclear cells

Cervical specimens were vortexed before the removal of cytobrush. They were filtered through a 70-μm nylon cell strainer (Becton Dickinson, San Diego, CA, USA) and centrifuged at 200 g for 10 min and the cell pellet was resuspended in PBS. Mononuclear cells were separated by Ficoll-Paque density gradient centrifugation. The mononuclear cells were counted on haemocytometer and samples containing less than one million cells/ml were excluded. The mononuclear cells were washed three times with PBS and suspended in RPMI-1640 (Sigma-Aldrich) containing 5% fetal calf serum (FCS) (PAA, Austria). Briefly, endocervical mononuclear cells were cultured in round-bottomed 96-well plates (5 × 10^4 ^cells/well) in a total volume of 200 μl and subsequently stimulated with cHSP60 (2 μg/ml) and cHSP10 (3 μg/ml) in triplicate. Phytoheamagglutinin (PHA 2 μg/ml) (Sigma) was used as a positive control mitogen in each experiment. Optimum concentrations of antigen and mitogen were determined in preliminary experiments as optimum concentrations giving maximal proliferation post stimulation.

### Antibody assays

Cervical washes of patients and controls were assayed for presence of IgG antibodies to cHSP60 and cHSP10. Briefly, the proteins were bound to the wells of a microtitre plate (1 μg/well) in carbonate buffer (14.2 mM Na_2_CO_3_, 34.9 mM NaHCO_3_, 3.1 mM NaN_3_, pH 9.5) and were incubated overnight at 4°C. After washing, wells were blocked with PBS-0.5% bovine serum albumin (BSA) at 37°C for 60 min; 100 μl of cervical washes were then added, and after incubation at 37°C for 120 min 100 μl of 1: 10 000 dilution of peroxidase-conjugated goat antibody to human IgG (Jackson Immunoresearch, Baltimore, MD, USA) was added to each well. After further incubation of 60 min at 37°C, the peroxidase substrate tetramethylene benzidine was added. The reaction was stopped with 0.5 M H_2_SO_4 _and the plates were read at 450 nm. Known positive and negative controls were always assayed in parallel to test samples. A positive sample was defined as one yielding an OD value that was at least 2 standard deviations (SD) above the mean value of known negative samples as mentioned earlier [30].

### Cytokine assays using ELISA

Quantification of IFN-γ, IL-10, TNF-α, IL-13 and IL-4 (ebiosciences, San Diego, CA, USA) in the supernatant of cervical mononuclear cell cultures of samples after 72 h stimulation with proteins was performed by commercially available ELISA kits, in accordance with the manufacturer's instructions. The minimum detectable cytokine concentrations for these assays were IFN-γ-(4 pg/ml), IL-10-(2 pg/ml), TNF-α-(4 pg/ml), IL-13-(4 pg/ml) and IL-4-(2 pg/ml).

### Statistical analysis

Since the distributions in cytokine production were not normal, differences between two groups were evaluated using Mann-Whitney *U *test. A comparison between two groups was made only when the Kruskal-Wallis test yielded a statistically significant result. Categorical variables were compared using the χ^2 ^test. Correlation was tested with Spearman's correlation coefficient.

## Results

### Study population

Cervical *C. trachomatis *infection was diagnosed by direct fluorescent assay (DFA)/PCR in 174 patients. Thirty-one of these patients were found to have bacterial vaginosis, or to be co-infected with either *Candida *sp., *T. vaginalis*, *M. hominis*, *U. urealyticum *or *N. gonorrhoeae *in the cervix and were thus excluded from the study. Ten *Chlamydia *positive patients were excluded, as the count of mononuclear cells in the cervical cells was less than 1 million cells/ml and epithelial cells were present. Based on diagnosis the women were divided into three groups. Group I (n = 39) comprised of uninfected healthy controls with no infertility problem; Group II (n = 63) comprised of *Chlamydia *positive women with no infertility problem; Group III (n = 70) comprised of *Chlamydia *positive women with infertility and who had laparoscopic or hysterosalpingographic evidence of tubal damage. Candidates were considered infertile if they had regular unprotected intercourse for at least 2 years without conception. The median ages of women in each group were comparable (Table 1).

**Table 1:** Prevalance of cHSP60 and cHSP10 specific antibodies in study population

**Groups**	**Age Median (range)**	**cHSP60-IgG**	**cHSP10-IgG**
		
		**Number (%)**	
**Group I (n = 39)**	25 (21–37)	3 (8)	2 (5)
**Group II (n = 63)**	27 (23–43)	15 (24)^a^	13 (21)^b^
**Group III (n = 70)**	29 (21–42)	35 (50)^c,e^	26 (37)^d,f^

(n) represents number of patients

^a^P = 0.04 as compared to Group I; ^b^P = 0.03 as compared to Group I; ^c^P < 0.0001 as compared to Group I; ^d^P < 0.0001 as compared to Group I; ^e^P = 0.002 as compared to Group II; ^f^P = 0.04 as compared to Group II where,

Group I-Healthy women with no infertility problem

Group II-Chlamydia positive women with no infertility problem

Group III-Chlamydia positive women with infertility

Categorical variables were compared using the χ^2 ^test

### Detection of antibodies

**Figure 1:**
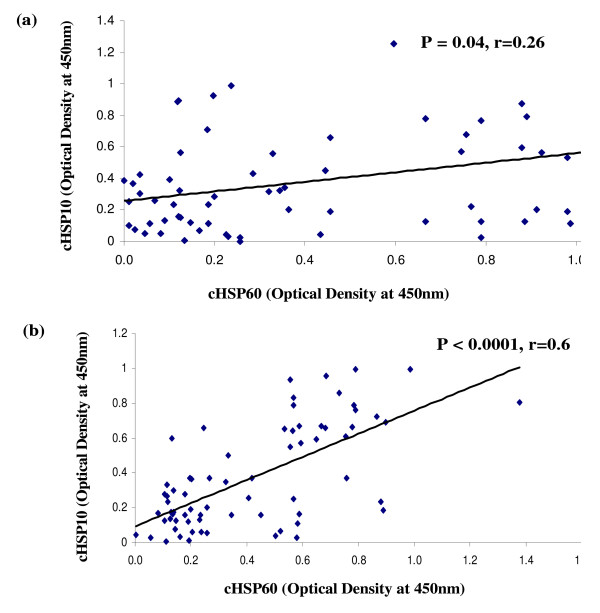
**Correlation of anti-cHSP60 and anti-cHSP10 IgG antibodies in cervical washes of patients**. Scatter plot showing the correlation between anti-cHSP60 and anti-cHSP10 IgG antibodies among patients groups (a) Group II and (b) Group III. A significant correlation was observed in Group II as well as Group III where, Group II (n = 63) – Chlamydia positive women with no infertility problem. Group III (n = 70) – Chlamydia positive women with infertility. Correlation was tested with Spearman's correlation coefficient

ELISA results showed that the prevalence was significantly higher for IgG antibodies to both cHSP60 (P = 0.04 & P < 0.0001) and cHSP10 (P = 0.03 & P < 0.0001) in Group II and III respectively as compared to Group I. In Group III the prevalence was significantly higher for IgG antibodies to both cHSP60 and cHSP10 as compared to Group II (P = 0.002 & P = 0.04 respectively) (Table 1). Correlation between cHSP60 and cHSP10 specific IgG antibodies were significant (P = 0.04, r = 0.26 & P < 0.0001, r = 0.6) in both Group II and Group III respectively (Figure 1a &1b).

### Cytokine production by stimulated cervical mononuclear cells

**Figure 2:**
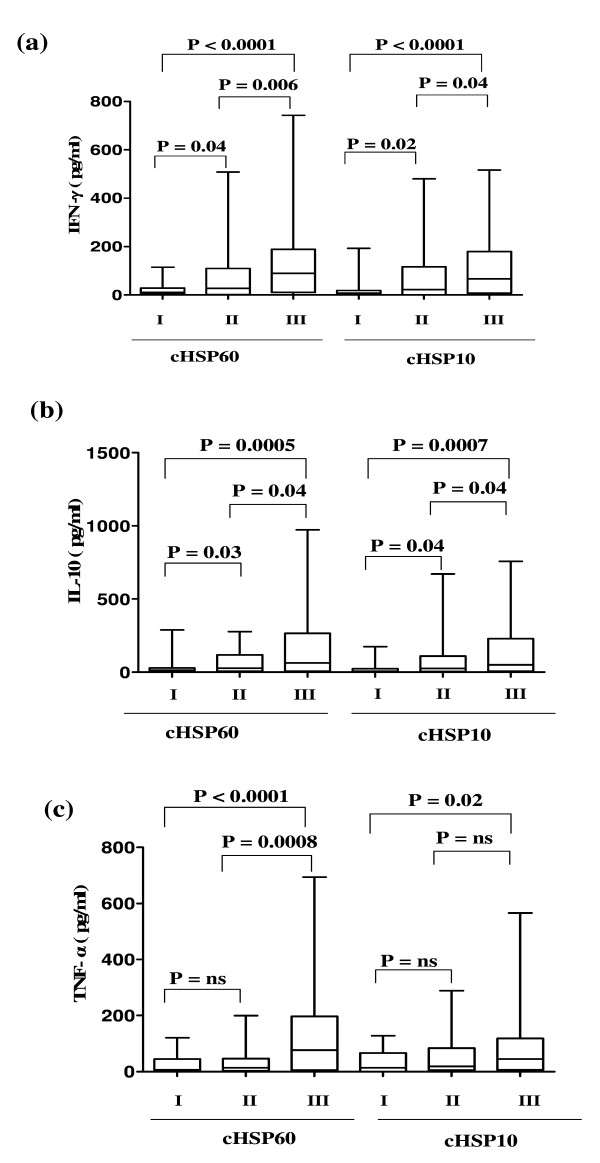
**Cytokine concentrations after stimulation with cHSP60 and cHSP10**. Box plot representing concentrations of (a) IFN-γ (b) IL-10 (c) TNF-α in supernatants of cervical mononuclear cells after stimulation with cHSP60 and cHSP10. A significant increase in levels of IFN-γ, IL-10 and TNF-α was observed after stimulation with cHSP60 and increased IFN-γ and IL-10 levels were observed after stimulation with cHSP10 in Group III as compared to Group I and Group II. The horizontal line in the middle of the box is the median value of the responses and the lower (upper) is the 25th (75th) percentile. I, II and III represent Group I, Group II and Group III respectively where, Group I (n = 39) – Healthy women with no infertility problem. Group II (n = 63) – Chlamydia positive women with no infertility problem. Group III (n = 70) – Chlamydia positive women with infertility. ns – Not significant. Mann-Whitney *U*-test was used for comparing two groups

IFN-γ levels were significantly higher after stimulation with both cHSP60 and cHSP10 in Group II (P = 0.04 & 0.02 respectively) and in Group III (P < 0.0001) as compared to Group I. Significantly higher levels of IFN-γ were observed after stimulation with both cHSP60 (P = 0.006) and cHSP10 (P = 0.04) in Group III when compared with Group II (Figure 2a). Similarly when IL-10 levels were compared significant difference was observed after stimulation with both cHSP60 and cHSP10 between Group II (P = 0.03 & 0.04 respectively) and Group III (P = 0.0005 & 0.0007 respectively) as compared to Group I and IL-10 was significantly higher (P = 0.04) in Group III as compared to Group II after cHSP60 and cHSP10 stimulation (Figure 2b).

TNF-α levels were significantly higher after stimulation with cHSP60 and cHSP10 (P < 0.0001 & P = 0.02 respectively) in Group III as compared to Group I whereas there was no significant difference (P = 0.07 & 0.25 respectively) in Group II as compared to Group I. The TNF-α levels were significantly higher (P = 0.0008) in Group III when stimulated with cHSP60 as compared to Group II. The TNF-α levels were higher although not significant (P = 0.1) in Group III when stimulated with cHSP10 as compared to Group II (Figure 2c).

IL-13 levels were low (close to the minimum detectable cytokine concentrations) and no significant difference was observed after stimulation with both cHSP60 and cHSP10 in any group. There was no detectable IL-4 production in any culture supernatant after stimulation with both cHSP60 and cHSP10 (Data not shown).

IFN-γ and IL-10 levels after stimulation with both cHSP60 and cHSP10 were significantly correlated in Group III (P < 0.0001, r = 0.54 and P = 0.004, r = 0.33 respectively)

Median and range for cytokine concentration in all the groups after cHSP60 and cHSP10 stimulation are mentioned in Table 2.

**Table 2:** Concentration (pg/ml) of cHSP60 and cHSP10 specific cytokines in different groups.

**Groups**	**IFN-γ**		**IL-10**		**TNF-α**	
	
	**cHSP60**	**cHSP10**	**cHSP60**	**cHSP10**	**cHSP60**	**cHSP10**
	
	**Median (range) in pg/ml**					
**Group I (n = 39)**	11 (0–115)	8 (0–193)	15 (0–289)	8 (0–174)	6 (0–121)	14 (0–128)
**Group II (n = 63)**	28 (0–508)	24 (0–480)	27 (0–278)	26 (0–671)	14 (0–199)	19 (0–289)
**Group III (n = 70)**	90 (0–743)	67 (0–516)	63 (0–973)	52 (0–758)	77 (0–694)	45 (0–566)

(n) represents number of patients

Group I-Healthy women with no infertility problem

Group II-Chlamydia positive women with no infertility problem

Group III-Chlamydia positive women with infertility

Median and range for cytokine concentration after PHA stimulation were: IFN-γ [Group I: 22 (0–276), Group II: 87 (5–643), Group III: 120 (11–1049)]; IL-10: [Group I: 20 (0–366), Group II: 69 (7–823), Group III: 104 (5–669)]; TNF-α [Group I: 17 (0–114), Group II: 72 (0–445), Group III: 92 (0–382)]; IL-4 [Group I: 0 (0–2), Group II: 7 (0–25), Group III: 8 (0–18)]; IL-13 [Group I: 0.8 (0–5), Group II: 24 (0–86), Group III: 18 (0–63)].

There was high standard error for cytokines as one or two samples showed very high concentration of cytokines. However, these high values did not affect the median value as well as the significance of the results when data was analyzed excluding these values.

## Discussion

Disease stages developed upon infection with Chlamydia are mediated by the immune responses. Immunity to *C. trachomatis *HSP60 and HSP10 is associated more typically with the chronic upper genital tract infection than it is with acute infection of the lower genital tract [15,31].

In the present study we detected higher cHSP60 and cHSP10 specific IgG antibody responses in the cervical washes of infertile group as compared to fertile group. These results were consistent with the previous reports where recurrent infection group showed high prevalence of cHSP specific IgG and IgA antibodies [25]. We also found a significant correlation of cHSP60 and cHSP10 IgG antibodies suggesting that co-expression of cHSP60 and cHSP10 occurs at the site of infection too. This observation again adds to the previous reports suggesting cHSP10 is co-expressed with cHSP60 [32].

We also evaluated cHSP60 and cHSP10 specific cytokines in cervical mononuclear cells and found IL-10 levels were more prominent when stimulated with both cHSP60 and cHSP10 in the infertility group as compared with other groups. Our data is consistent with the previous studies in which cHSP60 specific higher IL-10 levels in pheripheral blood mononuclear cells (PBMCs) have been reported in infertile women [21]. Cohen et al had recently demonstrated that cHSP60 specific IL-10 production by PBMCs act as a risk factor for *C. trachomatis *infection in humans [22]. The enhanced levels of IL-10 may not be acting as anti-inflammatory mediator and might be involved in prolonging the infection by exerting immunostimulatory effects [33]. Overall, these results allow hypothesizing the role of IL-10 in fibrosis and tubal infertility. Indeed, IL-10 has been involved in fibrosis in several human diseases [34-36] and an association of fibrosis with cHSP60 and cHSP10 specific antibodies has been reported in infected animals [37].

We also evaluated IFN-γ and TNF-α levels and higher levels of both the cytokines were detected in infertility group after stimulation with cHSP60 and cHSP10. IFN-γ production has been identified as one of the main factors in protective immunity [38] and is also important in the development of chronic chlamydial infection [8]. IFN-γ delays the developmental cycle of Chlamydia so that chlamydial reticulate bodies persist longer, which might result in persistent unapparent infection and also, play a role in immunopathogenesis by promoting inflammatory damage and fibrosis [26]. In addition, increased levels of IFN-γ have been reported in the endocervical secretions of *C. trachomatis *positive infertile women [27]. TNF-α which displays antichlamydial properties [39], is also known to play an important role in the initiation of inflammatory response. In the mouse genital tract, infertility associated with endometriosis has been shown to be related to the production of TNF-α [40]. In human, both IFN-γ and TNF-α have been reported to be associated with infertility [41-43]. Proinflammatory cytokines are also known to drive the lipid peroxidation of the spermatozoa plasma membrane to levels that can affect the sperm fertility capacity [44]. In addition TNF-α and IFN-γ have effects on sperm motility, viability, membrane integrity and lateral head displacement, suggesting poor fertilizing potential of human spermatozoa during inflammatory conditions [45]. Previous evidence suggests that the concurrent immunization with cHSP60 switches the cytokine production of self HSP60 responding T cells to dominant production of proinflammatory IFN-γ [46] showing that chlamydial HSP60 can break the tolerance of autoreactive cell reactions and lead them to participate in the inflammatory reactions during chlamydial disease. Hence from the present study higher levels of both IFN-γ and TNF-α by cHSPs in infertility group may suggest their involvement in the immunopathological condition associated with the infertility.

In our study, IL-4 was undetectable and IL-13 levels were not significantly different in any group in contrast to previous report in which cHSP60 specific IL-13 in PBMCs has been reported to be associated with the protective response [22]. It may suggest that cHSP specific Th2 cytokines (IL-4 and IL-13) does not play any role in pathogenesis related to chlamydial infection.

In our results high levels of IL-10 may not suggest a Th2 response as studies showed that it is secreted by Th1 and Th2 cells as well as other cells [47,48]. Further it has been shown that in many chronic infections in human and experimental animals, CD4+ T cells can produce high levels of both IL-10 and IFN-γ [49]. Hence the production of high levels of both the cytokines, IL-10 and IFN-γ in the absence of significant levels of other Th2 cytokines, suggests that the cells secreting IL-10 are not Th2 cells but other cells.

Previous studies, suggested that immune sensitization to HSPs probably require prolonged exposure of them at elevated concentrations [50]. As for cHSP60, there have been reports that during repeated and severe *C. trachomatis *infection there is enhanced recognition of cHSP60 by circulating lymphocytes [18,51] and it has been shown that PBMCs from women with tubal factor infertility responded more frequently to cHSP60 antigen [21]. Hence the differential responses to cHSPs in chlamydia infected fertile and infertile women would be due to prolonged exposure to cHSPs in infertile cases.

We also did the correlation analysis of different cytokines produced against cHSP60 and cHSP10 and observed a positive correlation for IL-10 and IFN-γ levels in infertility group suggesting similar role of cHSP10 in pathology associated with the infertility as cHSP60. There are no studies to date on cHSP10 specific cell mediated immune responses and our data suggests that the cHSP10 specific immune responses may have crucial role in the immunopathological condition associated with the infertility. Reports on the immunogenicity of HSP10 antigens from other microbial pathogens suggest that the HSP10 family of proteins are capable of eliciting chronic inflammation and delayed hypersensitivity. In particular, HSP10 homologues of *Mycobacterium leprae *and *Mycobacterium tuberculosis *have been shown to stimulate T-cell responses [52,53].

Overall our results suggest that exposure to the chlamydial heat shock proteins could significantly affect mucosal immune function by modifying the release of cytokines leading to severe immunopathological conditions related to infertility.

## Conclusion

The intent of the study provides some perspective on the ways in which cHSPs may contribute to the disease process associated with chlamydial infections. From the present study it can be suggested that the enhanced HSP expression leads to antigen specific increase in IFN-γ, IL-10 and TNF-α at the site of infection by cervical mononuclear cells suggesting cHSPs may consequently contribute to the immunopathogenesis associated with the infertility. This study also points out that further research is warranted to more precisely define the potential contribution of cHSPl0 and other conserved chlamydial antigens to the immunopathologic process associated with chlamydial infection.

## Competing interests

The authors declare that they have no competing interests.

## Authors' contributions

AM, PS and RJ had participated in the design of the study. The experiments were carried out by PS and RJ. Data analysis was performed by PS. SS had helped in collection of samples. AM and SB helped to draft the manuscript; the manuscript was written by PS. All authors have read and approved the final manuscript.

## References

[B1] World Health Organization (WHO) (2001). Global prevalence and incidence of selected curable sexually transmitted infections: overview and estimates.

[B2] Patrick DM (1997). Chlamydia control: Components of an effective control strategy to reduce the incidence of *Chlamydia trachomatis*. Can J Human Sex.

[B3] Brunham RC, Peeling R, Maclean I, Kosseim ML, Paraskevas M (1992). *Chlamydia trachomatis *associated ectopic pregnancy: serologic and histologic correlates. J Infect Dis.

[B4] Toye B, Laferriere C, Claman P, Jessamine P, Peeling R (1993). Association between antibody to the chlamydial heat-shock protein and tubal infertility. J Infect Dis.

[B5] Dutta R, Jha R, Salhan S, Mittal A (2008). *Chlamydia trachomatis*-specific heat shock proteins 60 antibodies can serve as prognostic marker in secondary infertile women. Infection.

[B6] Beatty WL, Byrne GI, Morrison RP (1993). Morphologic and antigenic characterization of interferon gamma-mediated persistent *Chlamydia trachomatis *infection in vitro. Proc Natl Acad Sci USA.

[B7] Beatty WL, Morrison RP, Byrne GI (1994). Immunoelectron-microscopic quantitation of differential levels of chlamydial proteins in a cell culture model of persistent *Chlamydia trachomatis *infection. Infect Immun.

[B8] Beatty WL, Byrne GI, Morrison RP (1994). Repeated and persistent infection with *Chlamydia *and the development of chronic inflammation and disease. Trends Microbiol.

[B9] Witkin SS, Linhares I, Giraldo P, Jeremias J, Ledger WJ (2000). Individual immunity and susceptibility to female genital tract infection. Am J Obstet Gynecol.

[B10] Debattista J, Timms P, Allan J, Allan J (2003). Immunopathogenesis of *Chlamydia trachomatis *infections in women. Fertil Steril.

[B11] Neuer A, Lam KN, Tiller FW, Kiesel L, Witkin SS (1997). Humoral immune response to membrane components of *Chlamydia trachomatis *and expression of human 60 kDa heat shock protein in follicular fluid of in-vitro fertilization patients. Hum Reprod.

[B12] Ault KA, Statland BD, King MM, Dozier DI, Joachims ML, Gunter J (1998). Antibodies to the chlamydial 60 kilodalton heat shock protein in women with tubal factor infertility. Infect Dis Obstet Gynecol.

[B13] Spandorfer SD, Neuer A, LaVerda D, Byrne G, Liu HC, Rosenwaks Z, Witkin SS (1999). Previously undetected *Chlamydia trachomatis *infection, immunity to heat shock proteins and tubal occlusion in women undergoing in-vitro fertilization. Hum Reprod.

[B14] Betsou F, Sueur JM, Orfila J (1999). Serological Investigation of *Chlamydia trachomatis *Heat Shock Protein 10. Infect Immun.

[B15] LaVerda D, Albanese LN, Ruther PE, Morrison SG, Morrison RP, Ault KA, Byrne GI (2000). Seroreactivity to *Chlamydia trachomatis *Hsp10 correlates with severity of human genital tract disease. Infect Immun.

[B16] den Hartog JE, Land JA, Stassen FR, Kessels AG, Bruggeman CA (2005). Serological markers of persistent *C. trachomatis *infections in women with tubal factor subfertility. Hum Reprod.

[B17] Dadamessi I, Eb F, Betsou F (2005). Combined detection of *Chlamydia trachomatis *specific-antibodies against the 10 and 60-kDa heat shock proteins as a diagnostic tool for tubal factor infertility: Results from a case-control study in Cameroon. FEMS Immunol Med Microbiol.

[B18] Witkin SS, Jeremias J, Toth M, Ledger WJ (1994). Proliferative response to conserved epitopes of the *Chlamydia trachomatis *and human 60-kilodalton heat-shock proteins by lymphocytes from women with salpingitis. Am J Obstet Gynecol.

[B19] Kinnunen A, Molander P, Morrison R, Lehtinen M, Karttunen R, Tiitinen A, Paavonen J, Surcel HM (2002). Chlamydial heat shock protein 60-specific T cells in inflamed salpingeal tissue. Fertil Steril.

[B20] Debattista J, Timms P, Allan J, Allan J (2002). Reduced levels of gamma-interferon secretion in response to chlamydial 60 kDa heat shock protein amongst women with pelvic inflammatory disease and a history of repeated *Chlamydia trachomatis *infections. Chlamydia trachomatis.

[B21] Kinnunen A, Surcel HM, Halttunen M, Tiitinen A, Morrison RP, Morrison SG, Koskela P, Lehtinen M, Paavonen J (2003). *Chlamydia trachomatis *heat shock protein-60 induced interferon-gamma and interleukin-10 production in infertile women. Clin Exp Immunol.

[B22] Cohen CR, Koochesfahani KM, Meier AS, Shen C, Karunakaran K, Ondondo B, Kinyari T, Mugo NR, Nguti R, Brunham RC (2005). Immunoepidemiologic profile of *Chlamydia trachomatis *infection: importance of heat-shock protein 60 and interferon-gamma. J Infect Dis.

[B23] Tiitinen A, Surcel HM, Halttunen M, Birkelund S, Bloigu A, Christiansen G, Koskela P, Morrison SG, Morrison RP, Paavonen J (2006). *Chlamydia trachomatis *and chlamydial heat shock protein 60-specific antibody and cell-mediated responses predict tubal factor infertility. Hum Reprod.

[B24] Vats V, Agrawal T, Salhan S, Mittal A (2007). Primary and secondary immune responses of mucosal and peripheral lymphocytes during *Chlamydia trachomatis *infection. FEMS Immunol Med Microbiol.

[B25] Agrawal T, Vats V, Salhan S, Mittal A (2007). Mucosal and peripheral immune responses to chlamydial heat shock proteins in women infected with *Chlamydia trachomatis*. Clin Exp Immunol.

[B26] Rottenberg ME, Gigliotti-Rothfuchs A, Wigzell H (2002). The role of IFN-gamma in the outcome of chlamydial infection. Curr Opin Immunol.

[B27] Reddy BS, Rastogi S, Das B, Salhan S, Verma S, Mittal A (2004). Cytokine expression pattern in the genital tract of *Chlamydia trachomatis *positive infertile women – implication for T-cell responses. Clin Exp Immunol.

[B28] Joyee AG, Thyagarajan SP, Rajendran P, Hari R, Balakrishnan P, Jeyaseelan L, Kurien T, STD Study Group (2004). *Chlamydia trachomatis *genital infection in apparently healthy adult population of Tamil Nadu, India: a population-based study. Int J STD AIDS.

[B29] Bas S, Muzzin P, Ninet B, Bornand JE, Scieux C, Vischer TL (2001). Chlamydial serology: comparative diagnostic value of immunoblotting, microimmunofluorescence test, and immunoassays using different recombinant proteins as antigens. J Clin Microbiol.

[B30] Dutta R, Jha R, Gupta S, Gupta R, Salhan S, Mittal A (2007). Seroprevalence of antibodies to conserved region of *Chlamydia trachomatis *heat shock proteins 60 and 10 in women in India. Br J Biomed Sci.

[B31] Arno JN, Yuan Y, Cleary RE, Morrison RP (1995). Serologic responses of infertile women to the 60-kd chlamydial heat shock protein (hsp60). Fertil Steril.

[B32] Morrison RP, Su H, Lyng K, Yuan Y (1990). The *Chlamydia trachomatis *hyp operon is homologous to the groE stress response operon of Escherichia coli. Infect Immun.

[B33] Conti P, Kempuraj D, Kandere K, Di Gioacchino M, Barbacane RC, Castellani ML, Felaco M, Boucher W, Letourneau R, Theoharides TC (2003). IL-10, an inflammatory/inhibitory cytokine, but not always. Immunol Lett.

[B34] Martinez JA, King TE, Brown K, Jennings CA, Borish L, Mortenson RL, Khan TZ, Bost TW, Riches DW (1997). Increased expression of the interleukin-10 gene by alveolar macrophages in interstitial lung disease. Am J Physiol.

[B35] Yang X, Gartner J, Zhu L, Wang S, Brunham RC (1999). IL-10 gene knockout mice show enhanced Th1-like protective immunity and absent granuloma formation following *Chlamydia trachomatis *lung infection. J Immunol.

[B36] Sato S, Hasegawa M, Takehara K (2001). Serum levels of interleukin-6 and interleukin-10 correlate with total skin thickness score in patients with systemic sclerosis. J Dermatol Sci.

[B37] Higgins DP, Hemsley S, Canfield PJ (2005). Association of uterine and salpingeal fibrosis with chlamydial hsp60 and hsp10 antigen-specific antibodies in Chlamydia-infected koalas. Clin Diagn Lab Immunol.

[B38] Wang S, Fan Y, Brunham RC, Yang X (1999). IFN-gamma knockout mice show Th2-associated delayed-type hypersensitivity and the inflammatory cells fail to localize and control chlamydial infection. Eur J Immunol.

[B39] Shemer-Avni Y, Wallach D, Sarov I (1988). Inhibition of *Chlamydia trachomatis *growth by recombinant tumour necrosis factor. Infect Immun.

[B40] Darville T, Andrews CW, Rank RG (2000). Does inhibition of tumor necrosis factor alpha affect chlamydial genital tract infection in mice and guinea pigs?. Infect Immun.

[B41] Naz RK, Butler A, Witt BR, Barad D, Menge AC (1995). Levels of interferon-gamma and tumor necrosis factor-alpha in sera and cervical mucus of fertile and infertile women: implication in infertility. J Reprod Immunol.

[B42] Ng SC, Gilman-Sachs A, Thaker P, Beaman KD, Beer AE, Kwak-Kim J (2002). Expression of intracellular Th1 and Th2 cytokines in women with recurrent spontaneous abortion, implantation failures after IVF/ET or normal pregnancy. Am J Reprod Immunol.

[B43] Kwak-Kim JY, Chung-Bang HS, Ng SC, Ntrivalas EI, Manqubat CP, Beaman KD, Beer AE, Gilman-Sachs A (2003). Increased T helper 1 cytokine responses by circulating T cells are present in women with recurrent pregnancy losses and in infertile women with multiple implantation failures after IVF. Hum Reprod.

[B44] Martínez P, Proverbio F, Camejo MI (2007). Sperm lipid peroxidation and pro-inflammatory cytokines. Asian J Androl.

[B45] Estrada LS, Champion HC, Wang R, Rajasekaran M, Hellstrom WJ, Aggarwal B, Sikka SC (1997). Effect of tumour necrosis factor-alpha (TNF-α) and interferon-gamma (IFN-γ) on human sperm motility, viability and motion parameters. Int J Androl.

[B46] Yi Y, Yang X, Brunham RC (1997). Autoimmunity to heat shock protein 60 and antigen-specific production of interleukin-10. Infect Immun.

[B47] Yssel H, Malefyt RDW, Roncarolo MG, Abrams JS, Lahesmaa R, Spits H, de Vries JE (1992). IL-10 is produced by subsets of human CD4+ T cell clones and peripheral blood T cells. J Immunol.

[B48] Trinchieri G (2007). Interleukin-10 production by effector T cells: Th1 cells show self control. J Exp Med.

[B49] Trinchieri G (2001). Regulatory role of T cells producing both interferon gamma and interleukin 10 in persistent infection. J Exp Med.

[B50] Witkin SS, Askienazy-Elbhar M, Henry-Suchet J, Belaisch-Allart J, Tort-Grumbach J, Sarjdine K (1998). Circulating antibodies to a conserved epitope of the *Chlamydia trachomatis *60 kDa heat shock protein (hsp60) in infertile couples and its relationship to antibodies to *C. trachomatis *surface antigens and the *Escherichia coli *and human HSP60. Hum Repod.

[B51] Witkin SS, Jeremias J, Toth M, Ledger WJ (1993). Cell-mediated immune response to the recombinant 57-kDa heat shock protein of *Chlamydia trachomatis *in women with salpingitis. J Infect Dis.

[B52] Mehra V, Bloom BR, Bajardi AC, Grisso CL, Sieling PA, Alland D, Convit J, Fan XD, Hunter SW, Brennan PJ, Rea TH, Modlin RL (1992). A major T cell antigen of *Mycobacterium leprae *is a 10-kD heat-shock cognate protein. J Exp Med.

[B53] Launois P, N'Diaye MN, Cartel JL, Mane I, Drowart A, van Vooren JP, Sarthou JL, Huygen K (1995). Fibronectin-binding antigen 85 and the 10-kilodalton GroES-related heat shock protein are the predominant TH-1 response inducers in leprosy contacts. Infect Immun.

